# Duhem Model-Based Hysteresis Identification in Piezo-Actuated Nano-Stage Using Modified Particle Swarm Optimization

**DOI:** 10.3390/mi12030315

**Published:** 2021-03-17

**Authors:** Khubab Ahmed, Peng Yan, Su Li

**Affiliations:** 1 Key Laboratory of High-Efficiency and Clean Mechanical Manufacture, Ministry of Education, School of Mechanical Engineering, Shandong University, Jinan 250061, China; khubab@hotmail.com; 2 Atom Horizon Electric, Jinan 250061, China

**Keywords:** model identification, piezo-actuated nano-stage, particle swarm optimization, Dhuem model

## Abstract

This paper presents modeling and parameter identification of the Duhem model to describe the hysteresis in the Piezoelectric actuated nano-stage. First, the parameter identification problem of the Duhem model is modeled into an optimization problem. A modified particle swarm optimization (MPSO) technique, which escapes the problem of local optima in a traditional PSO algorithm, is proposed to identify the parameters of the Duhem model. In particular, a randomness operator is introduced in the optimization process which acts separately on each dimension of the search space, thus improving convergence and model identification properties of PSO. The effectiveness of the proposed MPSO method was demonstrated using different benchmark functions. The proposed MPSO-based identification scheme was used to identify the Duhem model parameters; then, the results were validated using experimental data. The results show that the proposed MPSO method is more effective in optimizing the complex benchmark functions as well as the real-world model identification problems compared to conventional PSO and genetic algorithm (GA).

## 1. Introduction

Piezoelectric actuators (PEAs) have been widely used in precision positioning [1], energy harvesting [2,3] and tracking applications owing to their advantages of fast dynamic response, high electrical mechanical coupling efficiency and high positioning accuracy. The major drawback of PEA is internal hysteresis nonlinearity [4], which deteriorates their position/tracking performance. Several differential equation-based models, such as the Bouc–Wen model [5] and the Duhem model [6], as well as operator-based models, such as the Preisach model [7], the Prandtl–Ishlinskii (PI) model [8], the Krasnosel–skiiPokrovskii (KP) model [9] and the Jiles–Atherton model [10], have modeled and characterized the hysteresis behavior of PEAs. The Duhem model is widely used to characterize the hysteresis nonlinearity in PEAs owing to its differential nature and ability to characterize the hysteresis-memory effect [11]. However, for nonlinear hysteresis systems such as PAEs, determining the parameters of the Duhem model is a challenging task, which limits its application. Several studies have attempted to develop different model identification techniques, e.g., parameter identification using the least-squares algorithm [12], identification of the Duhem model parameters using artificial neural networks [13] and a recursive least-squares online identification method [14,15]. Recently, various swarm-intelligence-based optimization algorithms [16] have been used for system identification, e.g., model identification using the artificial bee colony algorithm [17].

Particle swarm optimization (PSO) is a widely used swarm-intelligence-based optimization method [18,19,20,21,22]. However, a major drawback of PSO is that it tends to get trapped in local optima and is, therefore, unable to find a global optimal solution. Several variants of PSO have been presented in the literature to solve these problems, e.g., identification of the PI model is presented in [23,24,25] using modified PSO (MPSO) with improved global search properties. Khan proposed a mutation mechanism to improve the global search properties of PSO in [26]. Identification of the parameters of the asymmetric Bouc–Wen model using a modified slandered PSO algorithm is presented in [27,28,29,30]. Reference [31] proposed a distance term in classical PSO to improve its optimization ability to identify hysteresis in a Scott–Russell mechanism. A genetic algorithm-based particle swarm optimization identification algorithm is proposed in [29]. Furthermore, to deal with the local optima problem of traditional PSO, a chaos-optimization-based PSO algorithm has been proposed in [32,33]. The solution to local optima problem presented in most of the previous literature, is to relocate the swarm members further away from the local optima [23,24,25,26,27,28,29,31]. This relocation-based solution may be effective in general optimization problems; however, in model identification problems, each dimension is an independent search space rather than a collective multi-dimensional search space. In such cases, relocating a particle as a whole means relocating all its parameter estimates to an equal distance from the local optima, which may not serve the purpose of optimal parameter identification, considering that each parameter to be identified has a different range.

This paper presents modeling and identification of hysteresis in piezo-actuated nano-stage using the Duhem model. A modified particle swarm optimization (MPSO) is proposed that can effectively solve the optimization-based model identification problems. A special randomness operator is introduced in PSO scheme that can solve the local optima problem of the traditional PSO algorithm. Different benchmark functions are used to compare the optimization abilities of the proposed MPSO with that of traditional optimization algorithm, e.g., PSO and genetic algorithm (GA). The proposed MPSO is then used to identify the parameters of the Duhem-based hysteresis model of piezo-actuated nano-stage. Simulation and experimental results demonstrate the effectiveness of the proposed scheme compared with traditional PSO and GA. The proposed MPSO algorithm can effectively identify other differential equation-based multidimensional search space models as well, such as Bouc–Wen, etc. Although the proposed MPSO is specially designed for parameter identification purposes, it may be useful to solve other complex real-world optimization problems where search dimensions are not uniform such as the problem presented in references [34,35].

The rest of the paper is organized as follows. The Duhem mathematical model is presented in Section 2. Section 3 presents the parameter identification scheme. A comparison between traditional and proposed PSO algorithms is presented in Section 4. Experimental setup and results are presented in Section 5. Finally, conclusions are drawn in Section 6.

## 2. Hysteresis Modeling

### 2.1. Duhem Model for Hysteresis in PEA

**Figure 1:**
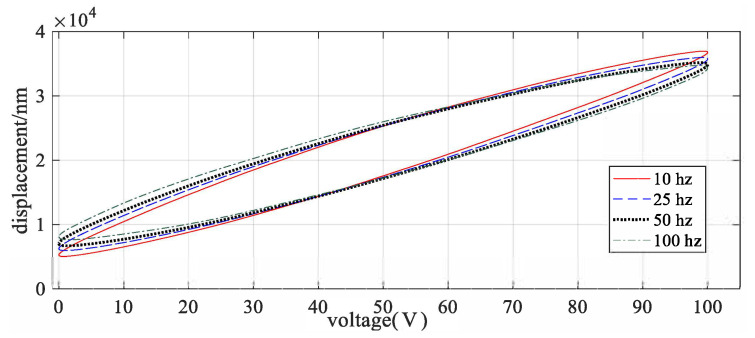
Hysteresis in the piezoelectric motion stage.

Due to the inherent property of piezoelectric materials, nonlinear hysteresis phenomenon is commonly observed in the input output relation of piezo driven motion stage, Figure 1. The Duhem model was first presented in 1986 to describe the hysteresis relationship for the ferromagnetic materials. The nonlinear relation between magnetic flux B(t) and magnetic field H(t) is described as:

(1) $$\dot{B}\left(t\right)=\beta \left|\dot{H}\left(t\right)\right|\left[\alpha H\left(t\right)-B\left(t\right)\right]+\alpha \gamma \dot{H}\left(t\right)$$

where α, β and γ are the parameters which define the shape and size of output hysteresis loop. For a single input single output (SISO) hysteresis system, the generalized Duhem model is given by:

(2) $$\begin{array}{c} y(t)=h(x(t),u(t)) \end{array}$$

(3) $$\begin{array}{c} \dot{x}\left(t\right)=f\left(x\left(t\right),u\left(t\right),g\left(\dot{u}\left(t\right)\right)\right),\;x\left(0\right)=x_{o},\;t\geq 0 \end{array}$$

where u(t):[0,inf)→R piecewise continuous $C^{1},g:R\to R^{r}$ is continuous and satisfies $g\left(0\right)=0,x\left(t\right):\left[0,\inf\right)\to R^{n}$ is absolutely continuous input, $f:R^{n}\times R\to R^{\left(n\times r\right)}$, $h:R^{n}\times R\to R$ and output y(t):[0,inf)→R are continuous.

For a piezo-actuated nano-stage, the linear dynamics y(t) can be described by the 2nd order system [13]:

(4) $$\begin{array}{c} \ddot{y}\left(t\right)+2\zeta \omega_{n}\dot{y}\left(t\right)+\omega_{n}^{2}y\left(t\right)=\omega_{n}^{2}h\left(t\right) \end{array}$$

where $\omega_{n}$ is the natural frequency of the actuator and ζ is the damping ratio. The quasi-static nonlinear hysteresis h(t) against the applied input voltage u(t) is described by the following set of equations:

(5) $$\begin{array}{c} h(t)=du(t)-x(t) \end{array}$$

(6) $$\begin{array}{c} \dot{x}\left(t\right)=\alpha \left|\dot{u}\left(t\right)\right|\left(\gamma u\left(t\right)-x\left(t\right)\right)+\beta \dot{u}\left(t\right) \end{array}$$

The model presented in (5) and (6) can be discretized as follows:

(7) $$\begin{array}{cc} \; & x(k)=x(k-1)+\alpha T|u(k-1)-u(k-2)|\\ \; & \left[\gamma u(k-1)-x(k-1)]+\beta T(u(k-1)-u(k-2)\right) \end{array}$$

(8) $$\begin{array}{c} h(k)=du(k)-x(k) \end{array}$$

where *T* is the sampling time period. The amplitude and shape of the hysteresis loop are dependent on the parameters α, β and γ whereas *d* is the piezoelectric coefficient. The following will describe the effect of parameters on the output hysteresis curve.

### 2.2. Effect of Parameters on the Duhem Model

**Figure 2:**
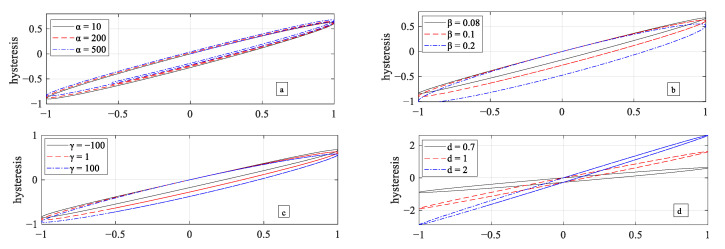
Change in hysteresis curve of the Duhem model with change in different parameters.

In this section, the Duhem model is simulated to study the effect of each parameter on the model’s output. This will help in determining the range of the parameters for the identification process, hence improving the optimization efficiency. A sinusoidal input u(k)=2sin(k) is selected for simulation. Loop shaping parameters of the Duhem model, α, β, γ and *d*, are chosen to analyze the effect of parameters on the output hysteresis curve. During this analysis, each parameter is varied one by one and keeping the others constant. The model presented in (7) and (8) is simulated in Matlab and the relation between hysteresis output h(k) and input voltage u(k) is shown in Figure 2. The following conclusions were drawn from this analysis,

1. From Figure 2a, it is observed that the increase in the value of α causes an increase in the output hysteresis and the overall hysteresis curve moves upwards.
2. As seen in Figure 2b,c, the increase in the values of β and γ results in the increase in the width of the hysteresis curve as well as a downward movement in hysteresis curve is observed. Although the effect of change in both β and γ is very similar on output hysteresis, output hysteresis is much more sensitive to a small change in β as compared to γ.
3. The effect of change in parameter *d* on output hysteresis is shown in Figure 2d. A clockwise movement in the hysteresis curve can be observed with the increase in the value of *d*.

The above analysis shows that the effect of change in parameters on the Duhem-based hysteresis curve is complex which makes the parameter identification a difficult task. The next section describes the parameter identification scheme.

## 3. Parameter Identification Method

### 3.1. Modeling of Optimization Problem

The parameters of the Duhem model affect the model behavior in an indirect way; in reality, they do not have any physical meaning. Identification of such systems is a challenging task. Thus, an optimization-based identification method is proposed in this research.

The first step is to model the identification problem in (7) and (8) into an optimization problem. Let *P* be the set of parameters to be identified such that:

(9) $$\begin{array}{c} P_{min}\leq P\leq P_{max} \end{array}$$

(10) $$\begin{array}{c} \left[\begin{array}{c} \alpha_{\min}\\ \beta_{\min}\\ \gamma_{\min}\\ d_{\min} \end{array}\right]\leq \left[\begin{array}{c} \alpha \\ \beta \\ \gamma \\ d \end{array}\right]\leq \left[\begin{array}{c} \alpha_{\max}\\ \beta_{\max}\\ \gamma_{\max}\\ d_{\max} \end{array}\right] \end{array}$$

where $P_{min}$ and $P_{max}$ are the set of minimum and maximum possible values of parameters. Here, the objective function J(P) is defined to formulate the optimization problem.

(11) $$\begin{array}{c} J\left(P\right)=\left[Y\left(k\right)-\hat{Y}\left(k|P\right)\right]{\left[Y\left(k\right)-\hat{Y}\left(k|P\right)\right]}^{T} \end{array}$$

With parameters subjected to constraints defined in (10), the minimization of J(P) is an optimization problem for parameter identification. Y(k) is the reference time history which will be obtained experimentally and $\hat{Y}\left(k|P\right)$ is the augmented time history from the model to be identified. The objective function is formulated by minimizing the error between reference output Y(k) and the experimentally obtained data $\hat{Y}\left(k|P\right)$ [31].

### 3.2. Particle Swarm Optimization

PSO is a swarm intelligence-based optimization method, which consists of a group of randomly placed swarm particles (possible solutions). Swarm particles explore new solutions within the search space by moving to newer locations based on past experience of individual particles and the overall swarm.

Let *m* be the number of particles in the swarm in a *D* dimensional search space (*D* is the number of parameters in vector *P*) (9). Each particle in the swarm is characterized in the search space by its position $x_{k+1}^{i}=\left(x_{k}^{i,1},x_{k}^{i,2}\ldots x_{k}^{i,D}\right)$ and velocity $v_{k+1}^{i}=\left(v_{k}^{i,1},v_{k}^{i,2}\ldots v_{k}^{i,D}\right)$, where *k* is the iteration number and *i* is the particle number. Individual particle’s personal best and the global best optima are represented in vectors $pb_{k+1}^{i}=\left(pb_{k}^{i,1},pb_{k}^{i,2}\ldots pb_{k}^{i,D}\right)$ and $gb_{k+1}^{i}=\left(gb_{k}^{i,1},gb_{k}^{i,2}\ldots gb_{k}^{i,D}\right)$, respectively. The equation of motion for the particle *i* at the *k*th iteration is defined as [19]:

(12) $$\begin{array}{c} x_{k+1}^{i}=x_{k}^{i}+v_{k+1}^{i} \end{array}$$

(13) $$\begin{array}{c} v_{k+1}^{i}=\mu v_{k}^{i}+c_{1}r_{1}\left(pb_{k}^{i}-x_{k}^{i}\right)+c_{2}r_{2}\left(gb_{k}-x_{k}^{i}\right) \end{array}$$

where μ is the inertial factor, $r_{1}$ and $r_{2}$ are random numbers between 0 and 1, $c_{1}$ is the cognitive factor that assigns the weight to the personal performance of each particle and $c_{2}$ is the factor which assigns the weight to swarm performance on the individual particle.

### 3.3. Modified Particle Swarm Optimization (MPSO)

**Figure 3:**
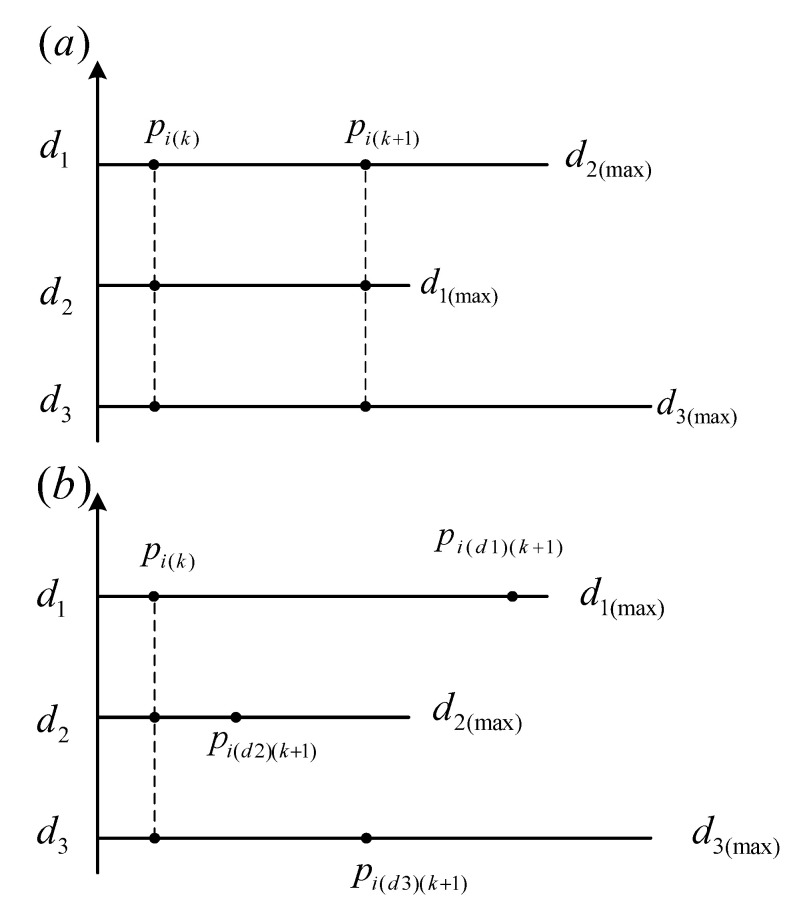
(**a**) Simple relocation of swarm particles and (**b**) the proposed modified particle swarm optimization (MPSO) bas relocation.

Ever since its development, PSO has been widely used in many applications to solve optimization problems. The main problems with PSO are its slow convergence rate and trapping into local minima. The latter is more critical while dealing with the model identification application. This paper presents a novel modified particle swarm optimization method which can deal with local optima and the slow convergence problem of PSO. The main idea is to reposition the swarm particles randomly in each search dimension such that the particles, which were previously confined to a local optima, get scattered all over the search space. The swarm particle relocation scheme presented here is different from previous modifications of PSO presented in literature because randomness is added to every search dimension of each particle separately. This makes the proposed MPSO more effective for model identification related optimization problems. Figure 3 demonstrates the difference between simple particle relocation and the effect of proposed randomeness operator.

Randomness operator *z* is defined for each search dimension that can relocate the swarm from local optima neighborhood to all over the search space. Consider a swarm with *m* particles moving in *n* dimensions for a minimization problem defined in (11). From (9), *P* is an n×1 matrix where *n* is the number of parameters to be identified in the optimization problem. In order to add randomness throughout the search space, each search dimension is divided into *n* portions. For the optimization problem defined in (11), *i*th particle’s randomness operator $z^{i(\alpha )}$, of parameter α from (10), at time step *k* is defined as:

(14) $$\begin{array}{c} z_{k}^{i(\alpha )}=\frac{\alpha_{r}}{n}r_{n}+\alpha_{\min}+\frac{\alpha_{r}}{n}q_{k}^{i(\alpha )} \end{array}$$

where $\alpha_{r}=\alpha_{\max}-\alpha_{\min}$ is the range of parameter α. $r_{n}$ is the random number between 0∼1, $q_{k}^{i(\alpha )}\subseteq Q$ is the quadrant number of the parameter α for range $\alpha_{r}$ at the time step *k*. The value of $q_{k}^{i(\alpha )}$ ranges from 0∼(n−1) and *Q* is an n×m matrix defined as:

(15) $$\begin{array}{c} Q=\left[\begin{array}{ccc} q_{11} & \cdots & q_{1m}\\ \vdots & \ddots & \vdots \\ q_{n1} & \cdots & q_{nm} \end{array}\right]. \end{array}$$

**Figure 4:**
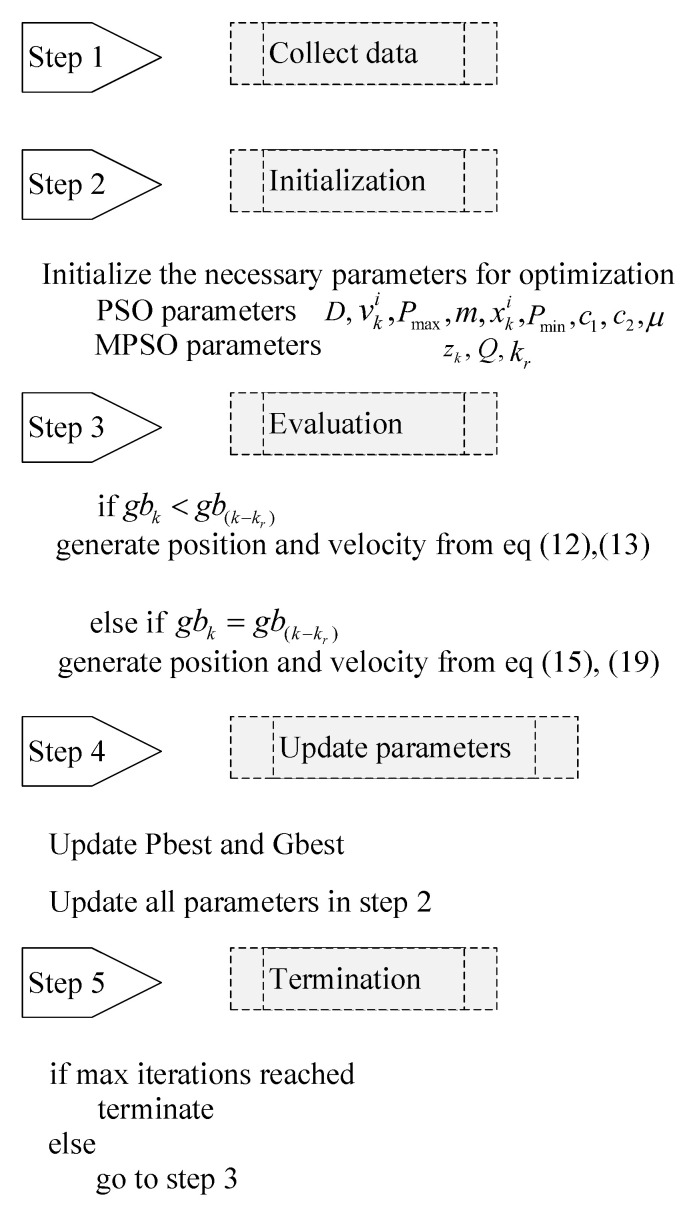
Flowchart of modified particle swarm optimization (MPSO).

If global best gb has not changed after $k_{r}<k_{max}$ number of iterations, then *Q* is generated to compute a new randomness factor *z* which is then replaced by swarm positions. From Equation (14):

(16) $$\begin{array}{c} z_{k}^{i(\beta )}=\left[r_{n}+q_{k}^{i(\beta )}\right]\frac{\beta_{r}}{n}+\beta_{\min} \end{array}$$

(17) $$\begin{array}{c} z_{k}^{i(\gamma )}=\left[r_{n}+q_{k}^{i(\gamma )}\right]\frac{\gamma_{r}}{n}+\gamma_{\min} \end{array}$$

(18) $$\begin{array}{c} z_{k}^{i(d)}=\left[r_{n}+q_{k}^{i(d)}\right]\frac{d_{r}}{n}+d_{\min} \end{array}$$

in general form

(19) $$\begin{array}{c} Z_{k}^{i}=\left[r_{n}+Q\right]\frac{P_{r}}{n}+P_{\min} \end{array}$$

where $z_{k}^{i(\alpha )}\subseteq Z_{k}^{i},P_{r}=P_{\max}-P_{\min}$ is the range matrix of parameters. Pseudocode of the proposed MPSO is presented in Figure 4.

## 4. Comparison with Conventional Optimization Algorithms

This section presents the performance analysis of the proposed MPSO compared with the traditional optimization algorithms such as GA and PSO. The first step is to define a suitable testbed that can generate the qualitative performance results for the optimization algorithms. A set of standard benchmark functions with different characteristics are usually employed to challenge the performance of an optimization algorithm and benchmark its abilities [36].

Benchmark functions used here can be categorized into unimodal and multimodal. Unimodal benchmark functions have only one global optimum and no local optima, thus they can be used to check the optimization/convergence speed of the algorithm and exploitative behavior. On the other hand, local optima avoidance and explorative abilities of an optimization algorithm can be tested by multimodal benchmark functions, the reason being these benchmark functions have many local optima along with one global optimum. Table 1 shows the details of the objective benchmark functions used in this work.

**Figure 5:**
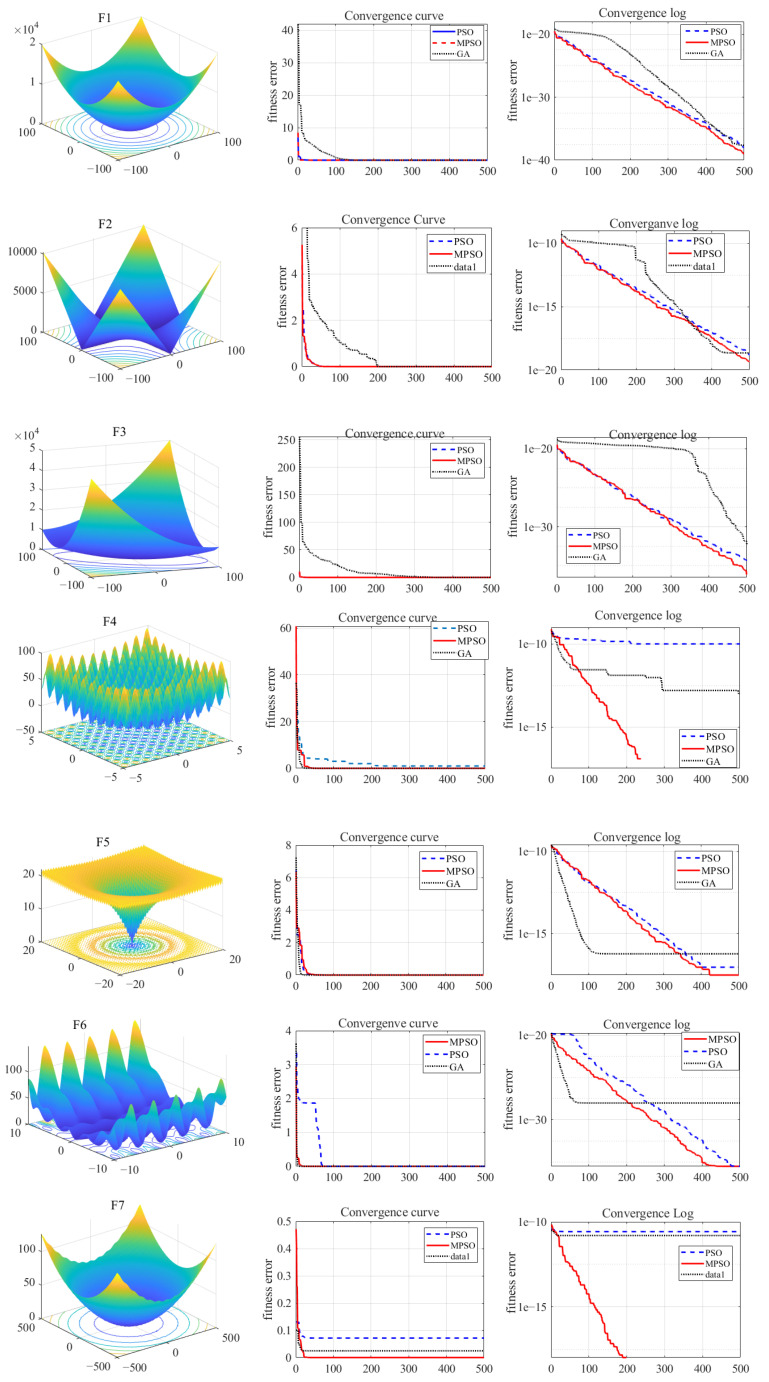
Performance analysis of genetic algorithm (GA), PSO and MPSO optimization on unimodal (**F1**–**F3**) and multimodal (**F4**–**F7**) benchmark functions.

A total of 7 benchmark functions are used to compare the optimization abilities of MPSO with traditional PSO and GA. Fifteen trials were run on each of the 7 benchmark functions using GA, PSO and MSPO to compare their performance. Figure 5 shows the shape of benchmark functions used and convergence trajectory (best among 15 trials) of GA, PSO and MPSO (minimization problem is considered here). In the case of unimodal benchmark functions, PSO and MPSO show similar performance (although MPSO performs slightly better)—the reason being there were no local optima present. On the other hand, while optimizing the multimodal benchmark functions, MPSO outperformed traditional PSO and GA. MPSO happened to converge faster in the presence of local optima. Table 2 shows average, standard deviation and best performance values of GA, PSO and MPSO against all the benchmark functions. From all of these results, it is clear that MPSO performs much better than the traditional GA and PSO algorithms in multi-dimensional optimization problems especially in the presence of local optima in the search space.

## 5. Parameter Identification and Experimental Validation

The Duhem model, presented in Section 2, consists of a nonlinear hysteresis model presented in (7) and (8) and linSectionear dynamic part (4). Here, the traditional PSO and proposed MPSO methods are utilized to determine the parameters of the Duhem model for nonlinear hysteresis. The following presents the experimental setup for the model identification process.

### 5.1. Experimental Setup

**Figure 6:**
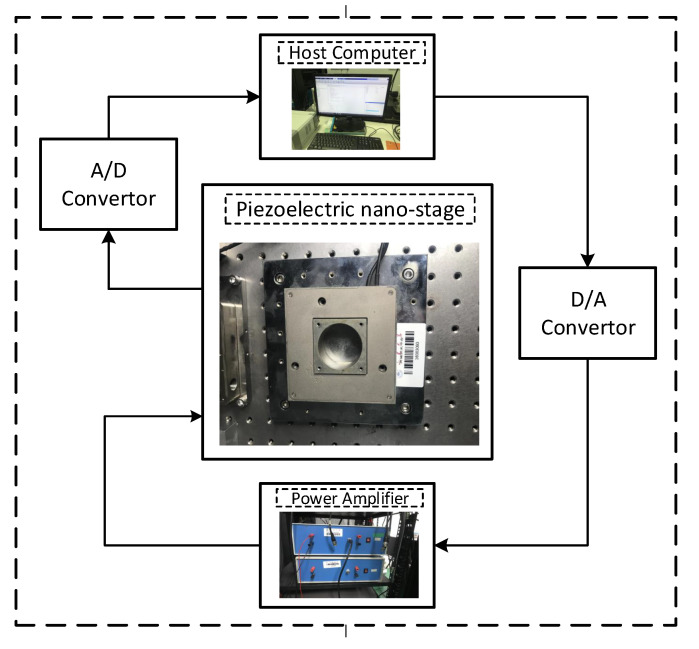
Experimental setup, piezo-actuated nano-stage connected to power amplifier and host computer with Matlab Realtime target, via 16 bit D/A and A/D, respectively.

The experimental setup configuration is shown in Figure 6. The piezoelectric actuator (NAC2014−H20 that features unloaded resonant frequency up to 52 kHz and unipolar driving voltage up to 150V) was attached to a motion stage. Matlab Simulink/xPc Target R2020a was used to generate the sinusoidal input signal u(k)=sin(2nft)+b. A 16bit resolution DAC interface of NI6259 was used to provide the input signal to the high bandwidth voltage amplifier. The motion stage (mounted on an air-floatation platform) was actuated in single DOF with the amplified signal and the output displacement was measured by MicroE systems Mercury II 6000 series linear encoder with 1.2 nm resolution and maximum speed of 61 mm/sec. A PCI6259 card was used for data acquisition, sampling rate of the experiment was set to 20 kHz. Necessary data for identification of the Duhem model was collected with the experimental setup. The model identification process is illustrated in the next sub-section.

### 5.2. Identification of Hysteresis Model

For parameter identification of the quasi-static nonlinear part of the Duhem model, we obtain input/output data experimentally at a frequency of the 1 Hz sine wave of 60 V amplitude. For positive output displacement, input waveform is kept positive. Next, general parameters of PSO and MPSO are defined. For the sake of fair comparison, both algorithms are initialized with the same basic parameters, i.e., total population of 50, inertial parameter *w* = 0.65 max iterations 500 and acceleration constants $c_{1}=c_{2}=2.05$. Iteration factor $k_{r}$ was set to 25 iterations for MPSO.

With the same experimental data, both MPSO and PSO were run 30 times each to identify the Duhem model for the optimization problem defined in (11). The best performance of MPSO was obtained at α=1.2324,β=−0.0058,γ=−4.7642,d=7.3694 and for PSO α=1.3258,β=−0.0053,γ=−5.1380,d=7.0152. The results of PSO and MPSO are shown in Table 3. It is clear that the proposed MPSO algorithm performs better than traditional PSO in model identification and convergence to global optima. In addition, a lesser number of iterations are taken by MSPO to converge to an optimum value.

### 5.3. Experimental Results

**Figure 7:**
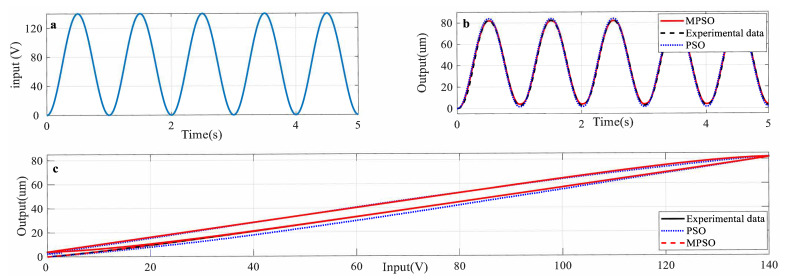
(**a**) Input excitation to the piezoelectric actuator (PEA) actuator, (**b**) predicted and measured output results, (**c**) hysteresis output predicted and measured results.

Figure 7a shows the input excitation to the piezoelectric actuator. With the identified parameters by MPSO described above, the predicted output of piezo-actuated motion-stage can be generated using (7) and (8). Figure 7b,c shows that the predicted output and hysteresis curve from the identified Duhem model are consistent with the experimentally obtained ones.

**Figure 8:**
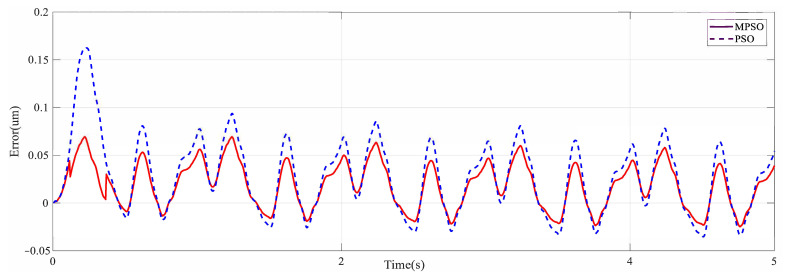
Output displacement error of PSO and MPSO.

**Figure 9:**
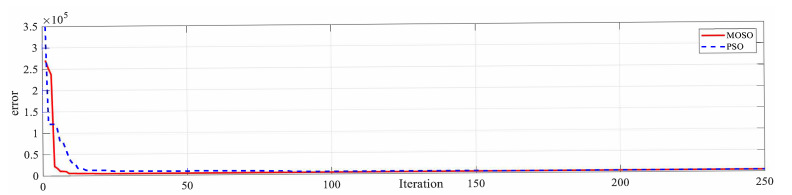
Convergence curve.

**Figure 10:**
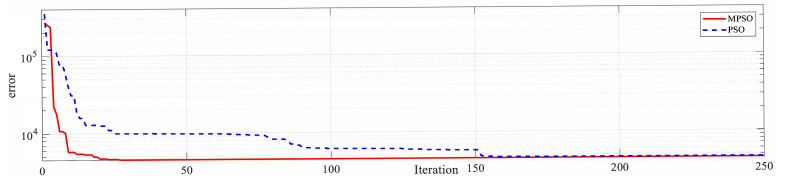
Convergence log.

Both PSO and MPSO successfully identify the Duehm model from experimental data. The superior model identification abilities of MPSO are evident from Figure 8, Figure 9 and Figure 10. Displacement errors from experimental results are shown in Figure 8 where the Duhem model is identified by both MPSO and PSO. It can be seen that the model identified by MPSO performs better as both average and maximum errors are on the lower side as compared to the one identified by traditional PSO.

**Figure 11:**
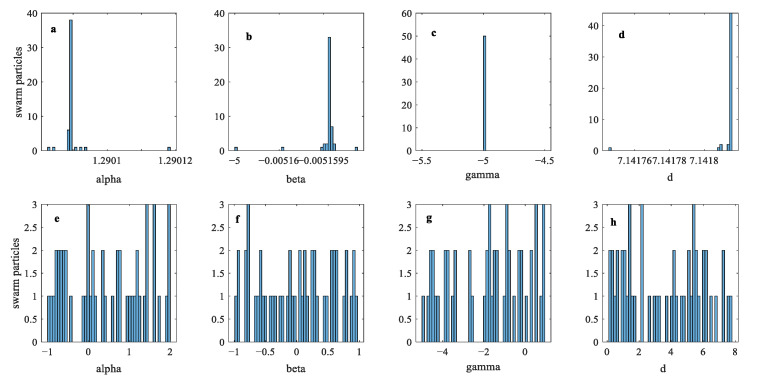
Swarm distribution before the randomness operator (**a**–**d**) and after the application of the randomness operator (**e**–**h**).

Convergence characteristics of MPSO and PSO are shown in Figure 9 and Figure 10, and it can be seen that MPSO is superior to traditional PSO in avoiding the local optima problem. In the current example of the Duhem model identification, MPSO encountered local minima at iteration 174 and the randomness operator from (19) comes into play and distributes swarm particles evenly in each search dimension. A comparison between swarm locations in the search space for parameters α,β,γ and *d* at the local minima condition and after application of MPSO’s randomness operator is shown in Figure 11a–d and Figure 11e–h, respectively.

**Figure 12:**
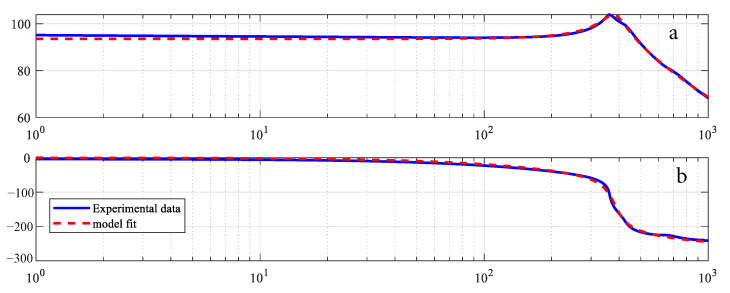
Frequency response (**a**) Magnitude (**b**) Phase.

**Figure 13:**
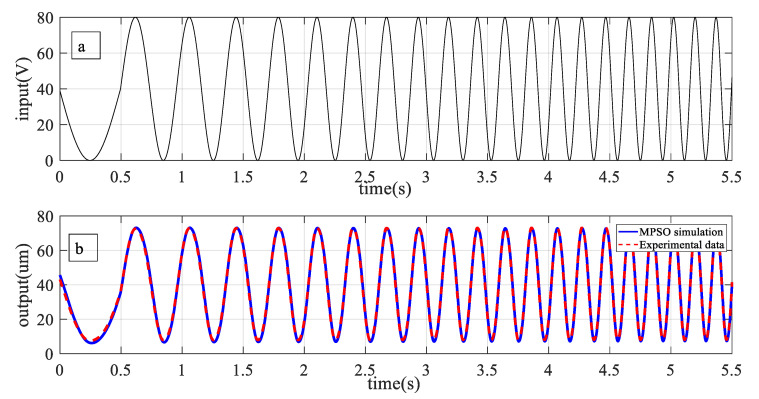
Performance validation of the overall model, measured and predicted output (**a**) input signal with varying frequency (**b**) output response.

**Figure 14:**
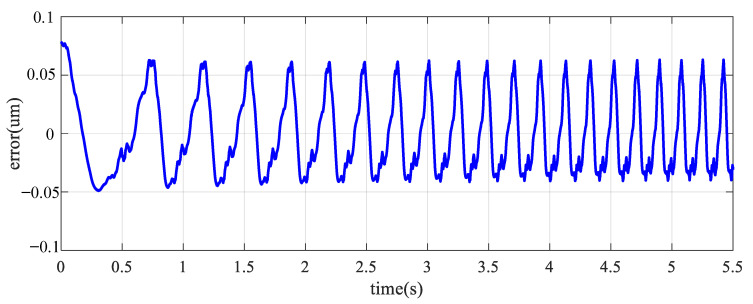
Error in overall model.

The overall behavior of the piezoelectric actuator can be described by combining the identified hysteresis submodel with the 2nd order dynamical submodel presented in (4). Frequency response method is used to identify the dynamic model. Figure 12 shows that the first response is at 277 Hz. For model validation, the piezo-actuated nano-stage was excited with a variable frequency signal (1 Hz–6 Hz), Figure 13a. Measured and predicted outputs, Figure 13b, show that the model output follows the experimental data with high precision. From the error plot shown in Figure 14, maximum error is 0.068 μm. The effectiveness of the proposed MPSO-based model identification method is further validated by this experiment, as the measured and predicted curves are consistent with each other for the overall model.

## 6. Conclusions

This paper describes the modeling and identification of the hysteresis behavior of piezo-actuated nano-stage using the Duhem model. The identification problem was first modeled into an optimization problem. A novel MPSO was specially designed to solve the model-identification related optimization problems. The proposed MPSO has superior model identification properties than traditional GA and PSO because it acts on each search dimension independently. This makes MPSO more effective in solving real time non-convex optimization problems with many local optima. The effectiveness of the proposed method was tested using different benchmark functions. The results showed that the proposed MPSO outperforms traditional GA and PSO algorithms when the search space is complex and contains many local optima. Finally, the proposed MPSO and PSO algorithms were used for identifying the Duhem model-based hysteresis in PEAs. The maximum displacement error between the proposed MPSO model and experimental data is 0.063 μm, whereas for traditional PSO, it is 0.1625 μm; this evidence shows that the proposed MPSO performs better in real-world model identification problems.

In the future, the performance of the proposed MPSO can be improved by introducing the objective functions designed specifically by utilizing its correlation with constraints of real-world problems.

## Figures and Tables

**Table 1:** Benchmark functions.

Function	Dim	Range	f (min)
$F1\left(x\right)=\sum_{i=1}^{n}x_{i}^{2}$	5	[−100, 100]	0
$F2\left(x\right)=\sum_{i=1}^{n}\left|x_{i}\right|+\prod_{i=1}^{n}\left|x_{i}\right|$	5	[−100, 100]	0
$F3\left(x\right)=\sum_{i=1}^{n}{\left(\sum_{j=1}^{n}x_{j}\right)}^{2}$	5	[−100, 100]	0
$F4\left(x\right)=\sum_{i=1}^{n}\left[x_{i}^{2}-10\cos\left(2\pi x_{i}\right)+10\right]$	5	[−5.12, 5.12]	0
$F5\left(x\right)=-20\exp\left(-0.2\sqrt{\frac{1}{n}\sum_{i=1}^{n}x_{i}^{2}}\right)$	5	[−20, 20]	0
$-\exp\left(\frac{1}{n}\sum_{i=1}^{n}\cos\left(2\pi x_{i}\right)\right)+20+e$
$F6\left(x\right)=\frac{\pi }{n}\{10\sin\left(\pi y_{1}\right)+\sum_{i=1}^{n-1}{\left(y_{i}-1\right)}^{2}$	5	[−100, 100]	0
$\left[1+10\sin^{2}\left(\pi y_{i+1}\right)\right]+{\left(y_{n}-1\right)}^{2}\}$
$+\sum_{i=1}^{n}u\left(x_{i},10,100,4\right)$
$F7\left(x\right)=\frac{1}{4000}\sum_{i=1}^{n}x_{i}^{2}-\prod_{i=1}^{n}\cos\left(\frac{x_{i}}{\sqrt{i}}\right)+1$	5	[−500, 500]	0

**Table 2:** Performance comparison between traditional optimization and MPSO algorithms.

Function		GA	PSO	MPSO
	Slandered deviation	5.147E-36	3.09E-37	3.17E-37
**F1**	Average	6.146E-36	3.21E-37	4.58E-37
	Best	5.2694E-36	3.07E-37	1.76E-18
	Slandered deviation	5.64E-18	1.80E-18	2.81E-19
**F2**	Average	5.031E-18	3.81E-18	4.58E-19
	Best	4.24E-18	2.41E-18	1.76E-19
	Slandered deviation	2.858E-26	1.88E-29	7.33E-32
**F3**	Average	2.658E-26	2.92E-29	1.11E-31
	Best	2.552E-26	1.29E-29	6.17E-33
	Slandered deviation	2.827E-6	0.430	0
**F4**	Average	2.5519E-6	0.746	0
	Best	1.038E-6	0	0
	Slandered deviation	5.586E-13	0	1.78E-15
**F5**	Average	4.805E-13	4.44E-15	2.07E-15
	Best	3.4195E-13	4.44E-15	8.88E-16
	Slandered deviation	9.394E-17	1.058E-31	9.713E-32
**F6**	Average	9.393E-17	1.136E-31	9.762E-32
	Best	9.276E-17	9.52E-32	9.4233E-32
	Slandered deviation	0.27175	0.01565583	0.009744069
**F7**	Average	0.26305	0.0456	0.012925
	Best	0.204612	0.032	0

**Table 3:** Model identification performance of PSO and MPSO.

Error Values					
	**std**	**avg**	**Best**	**Worst**	**avg Iteration**
PSO	0.3552	4617.3279	4614.4632	4619.2971	371
MPSO	0.8297	4531.3518	4529.7149	4534.3617	286

## Abbreviations

The following abbreviations are used in this manuscript:

PSO	Particle Swarm Optimization
MPSO	Modified Particle Swarm Optimization
PEA	Piezoelectric Actuator
